# Recent Insights in Barium-131 as a Diagnostic Match for Radium-223: Cyclotron Production, Separation, Radiolabeling, and Imaging †

**DOI:** 10.3390/ph13100272

**Published:** 2020-09-25

**Authors:** Falco Reissig, David Bauer, Martin Ullrich, Martin Kreller, Jens Pietzsch, Constantin Mamat, Klaus Kopka, Hans-Jürgen Pietzsch, Martin Walther

**Affiliations:** 1 Helmholtz-Zentrum Dresden-Rossendorf, Institut für Radiopharmazeutische Krebsforschung, Bautzner Landstraße 400, D-01328 Dresden, Germany; f.reissig@hzdr.de (F.R.); d.bauer@hzdr.de (D.B.); m.ullrich@hzdr.de (M.U.); m.kreller@hzdr.de (M.K.); k.kopka@hzdr.de (K.K.); h.j.pietzsch@hzdr.de (H.-J.P.); m.walther@hzdr.de (M.W.); 2 Fakultät Chemie und Lebensmittelchemie, Technische Universität Dresden, D-01062 Dresden, Germany

**Keywords:** barium-131, cyclotron production, radionuclide separation, macropa, small animal SPECT

## Abstract

Barium-131 is a single photon emission computed tomography (SPECT)-compatible radionuclide for nuclear medicine and a promising diagnostic match for radium-223/-224. Herein, we report on the sufficient production route ^133^Cs(*p*,*3n*)^131^Ba by using 27.5 MeV proton beams. An average of 190 MBq barium-131 per irradiation was obtained. The SR Resin-based purification process led to barium-131 in high radiochemical purity. An isotopic impurity of 0.01% barium-133 was detectable. For the first time, radiolabeling of the ligand macropa with barium-131 was performed. Radiolabeling methods under mild conditions and reaction controls based on TLC systems were successfully applied. Small animal SPECT/ computed tomography (CT) measurements and biodistribution studies were performed using [^131^Ba]Ba(NO_3_)_2_ as reference and ^131^Ba-labeled macropa in healthy mice for the first time. Biodistribution studies revealed the expected rapid bone uptake of [^131^Ba]Ba^2+^, whereas ^131^Ba-labeled macropa showed a fast clearance from the blood, thereby showing a significantly (*p* < 0.001) lower accumulation in the bone. We conclude that barium-131 is a promising SPECT radionuclide and delivers appropriate imaging qualities in small animals. Furthermore, the relative stability of the ^131^Ba-labeled macropa complex in vivo forms the basis for the development of sufficient new chelators, especially for radium isotopes. Thereby, barium-131 will attain its goal as a diagnostic match to the alpha emitters radium-223 and radium-224.

## 1. Introduction

**Figure 1:**
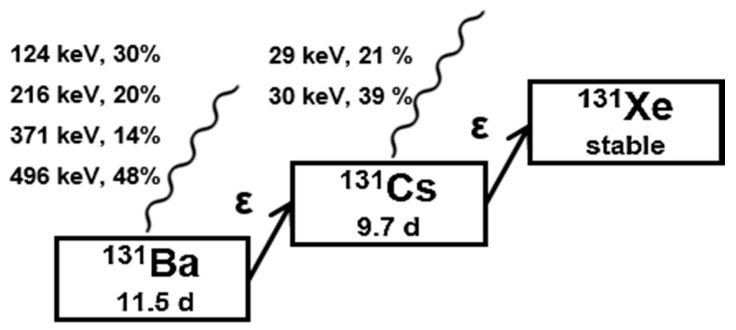
Decay scheme of barium-131.

The γ-emitter barium-131 (t_½_ = 11.5 d) decays via cesium-131 (t_½_ = 9.7 d) to stable xenon-131, each by electron capture (cf. Figure 1) [1]. In particular, the γ-energy 123.8 keV (30%) of the first decay is suitable for single photon emission computed tomography (SPECT) imaging. The calcimimetic behavior of free barium ions make barium-131 a useful long-term bone targeting radionuclide [2].

In the early 1970s, barium-131 has been thoroughly investigated as a potential bone targeting radiotracer by R. P. Spencer et al. [2,3,4] and J. Mahlstedt et al. [5], but no substantial benefits have been mentioned, comparing it to other already applicable radiotracers like [^18^F]F^−^ (t_½_ = 110 min) and ^99m^Tc-labeled (t_½_ = 6.0 h) bisphosphonates [6,7]. However, as part of current approaches to the therapy of bone cancer and bone metastases, this radionuclide has its significance in modern times.

Barium-131 possesses the suitable half-life of 11.5 d [1], thereby making it highly beneficial for potential diagnostic use in nuclear medicine [2,3]. Due to the similar chemistry [8] and pharmacological properties [9] of the elements barium and radium, barium-131 is particularly feasible as a diagnostic match to the therapeutic α-emitters radium-223 and radium-224. Radium-223 (t_½_ = 11.4 d) in the form of [^223^Ra]RaCl_2_ is the active component of Xofigo^®^ (Bayer, Berlin, Germany)—a drug which prolongs the survival of patients with bone metastases derived from castration-resistant prostate cancer (mCRPC) [10]. The α-emitter radium-224 (t_½_ = 3.6 d) was used for decades to treat the joint and bone disease ankylosing spondylitis (Morbus Bechterew; drug name: ^224^SpondylAT^®^, Eckert & Ziegler, Berlin, Germany) [11,12]. Currently, it is being investigated for a novel form of brachytherapy technique called diffusing alpha emitter radiation therapy (DaRT), where ^224^Ra-coated seeds are inserted intratumorally [13,14]. For present and future applications of radium-223 and radium-224, the radionuclide barium-131 functions as imaging analog providing bright prospects for long-term imaging, dosimetry planning, and therapy monitoring.

It is worth mentioning that the radioisotopes barium-135 m (t_½_ = 28.7 h) [2] and barium-133m (t_½_ = 38.9 h) [15] have been reported in the literature as potential bone tracers, as well. Both possess a shorter half-life than barium-131, but most importantly, they provide substantially worse properties for imaging. Barium-135m emits a γ-energy 268 keV (16%) and barium-133m at 276 keV (18%), respectively. In comparison with barium-131 (123.8 keV, 30%), this energy range leads to a higher inaccuracy in spatial resolution. Additionally, barium-133m decays to the long-lived radionuclide barium-133 (t_½_ = 10.5 a), which makes it disadvantageous as a medical radiotracer.

To meet the goal of establishing barium-131 as imaging radionuclide in target-specific radiopharmaceuticals, some obstacles have to be overcome. Barium-131 has the potential to be stably bound within any appropriate vehicle system, analogously as recently reported for radium; current studies are investigating the possibilities of incorporating radium ions into nanoparticles [16], e.g., based on hydroxyapatite [17,18]. Additionally, some approaches deal with the chelation of radium ions by organic ligands based on the calix [4] arene scaffold [19,20,21]. The chelator macropa, which is already under investigation as suitable chelator for actinium-225, could be—as recently reported [22,23,24]—particularly promising for heavy alkaline earth metals like radium. All data and conclusions of radium-approaches can be easily transferred to the γ-emitter barium-131, especially since barium studies have already been performed in function of a non-radioactive surrogate for radium [16,19,21].

The mother nuclide barium-131 can be used as a source for n.c.a. cesium-131 [25]. So far, the most common way of cesium-131 production is the neutron activation of barium-130 (natural abundance: 0.1%). When irradiated, barium-130 captures a neutron, becoming barium-131, which decays to cesium-131 [26]. However, an enriched barium-130 target is required, and still the production of long- and short-lived by-products cannot be impeded.

The daughter nuclide cesium-131 decays to stable xenon-131 by electron capture (cf. Figure 1) emitting photon radiation in the X-ray energy range (29–35 keV (73%)) [1]. Due to its optimal physical properties, cesium-131 is applied for brachytherapy of malignant tumors [27]. This radionuclide is primarily used, immobilized on the surface of seeds or needles, for the therapy of prostate cancer (trade name: Cesium Blu) [28]. In summary, barium-131 is not only interesting for the direct use in nuclear medicine, but also supports the development of new targeted alpha-particle therapy (TAT) approaches with radium-223 and radium-224, respectively.

**Figure 2:**
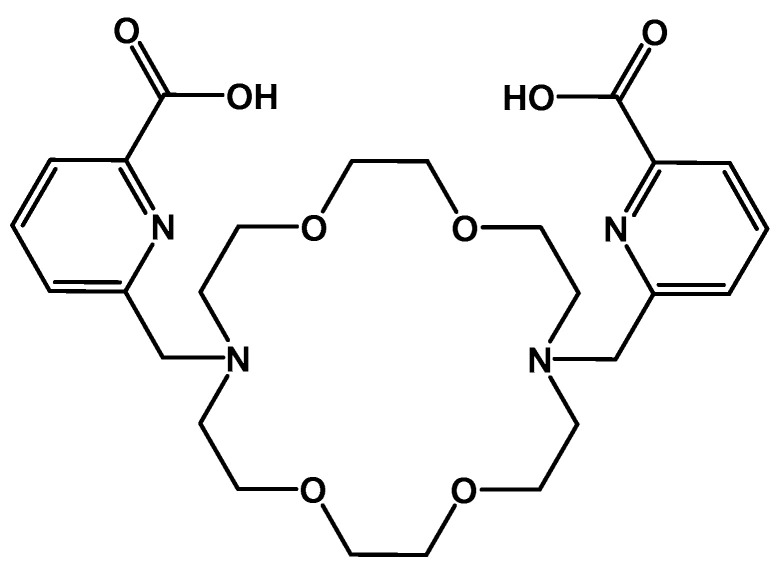
Structural formula of macropa.

In the work presented here, we aimed to establish a simple but sufficient procedure for the production and purification of n.c.a. barium-131 using the TR-FLEX cyclotron (ACSI), starting from a cheap cesium chloride target with natural monoisotopically occurring cesium followed by 27.5 MeV proton bombardment. The benefit of this method is yielding n.c.a. barium-131 with a relatively small contamination of barium-133 (0.01%; t_½_ = 10.5 a). This corresponds to the expected value due to the cross section of ^133^Cs(*p*,*n*)^133^Ba reaction is below 10% of the ^133^Cs(*p*,*3n*)^133^Ba reaction in the energy range between 26.7 MeV and 27.5 MeV. Additionally, an acceptable irradiation time (4 h) is required to produce an adequate level of activity (190 ± 26 MBq in 0.01 M HNO_3_). The in-house produced barium-131 was used for first labeling studies with the chelator macropa (cf. Figure 2), for initial in vivo-related phantom studies and, last but not least, small animal imaging trials with [^131^Ba]Ba(NO_3_)_2_ and ^131^Ba-labeled macropa in healthy mice.

## 2. Results and Discussion

### 2.1. Target Fabrication and Irradiation

After the careful adjustment of the beam intensity and the beam profile to the currently used conditions, the produced barium-131 shows reproducible activity values in the expected quantity. The used 80 mg of CsCl target material corresponds to an area density of 70 mg/cm^2^. The energy lost was calculated by SRIM (www.srim.org), and it is 840 keV within the CsCl. Hence, the used energy range of 27.5–26.7 MeV is slightly below the optimal energy window (29–28 MeV) due to the current design of the solid target unit with its energy degrader. The small layer thickness is determined by the low thermal conductivity of the cesium salt and best possible avoidance of the corresponding (*p*,*n*)- and (*p*,*2n*)-reactions to stable barium-132 and long-lived barium-133.

**Figure 3:**
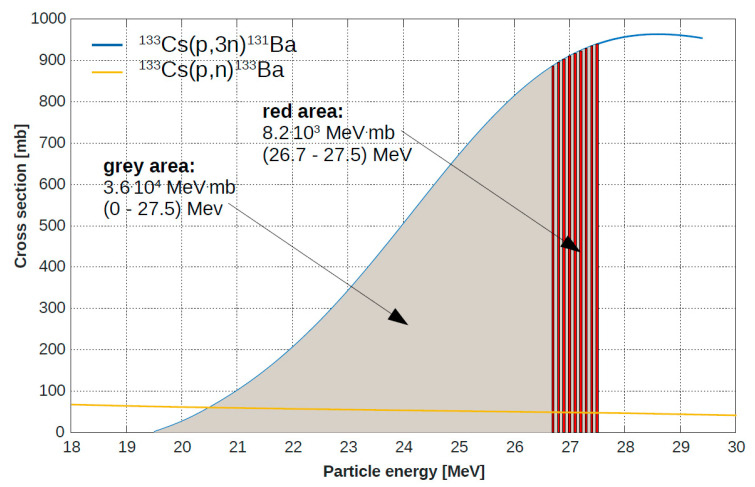
Cross section for the barium-131 production (blue line) and barium-133 (yellow line).

The saturation yield of the barium-131 production for a thick target is 9.1·10^3^ MBq/µA (https://www-nds.iaea.org/radionuclides/csp31ba0.html). A maximal activity of 1.4 GBq could be achieved with a thick target and the used irradiation parameters (4 h, 15 µA). To estimate the maximal producible activity with our target condition, we estimated the activity of our target thickness as follows. The cross section for the ^133^Cs(*p*,*3n*)^131^Ba reaction is shown in Figure 3. The thick target yield is based on the cross section of the complete energy range from 0 to 27.5 MeV. Only the area between 26.7 to 27.5 MeV is used for the nuclear reaction in the used target. Hence, the activity for a thick target has to be scaled by the ratio of the integral of the cross section between the used energy range and the whole energy range. A theoretical maximum activity of 320 MBq could be achieved with the used target and irradiation parameters using Equation (1).

(1) $$\frac{8.2\cdot 10^{3}\;\mathrm{MeV}\;\mathrm{mb}}{3.6\cdot 10^{4}\;\mathrm{MeV}\;\mathrm{mb}}\times 1400\;\mathrm{MBq}=320\;\mathrm{MBq}$$

An influence of moisture on the target was not observed in the short time between preparation and irradiation. Except darkening in the irradiated area, the pressed cesium salt shows no significant changes after irradiation. The dark color disappears immediately during the addition of nitric acid. For further irradiations, a second solid target system without energy degrader and another careful increase in intensity seems to be possible. The target current can be increased by a factor of 2 without increasing the power density on the target by using the 30° solid target holder. Hence, the amount of ^131^Ba is possible to be more than doubled within the same irradiation time.

### 2.2. Barium-131 Purification

**Figure 4:**
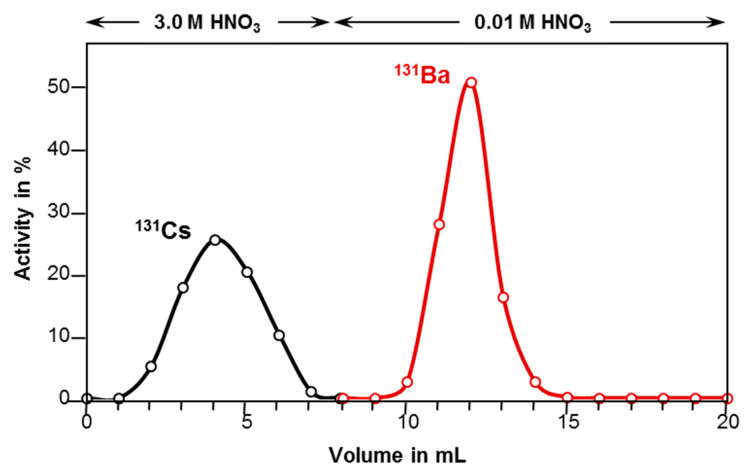
Elution profile of the barium-131 separation from a 80 mg CsCl target (6 mm × 100 mm Sr Resin column, gradient elution).

In average, a total barium-131 activity of (190 ± 26) MBq was obtained, as determined within four equal and independent irradiations analyzing the crude target solution. This corresponds to a saturation yield of (1.2 ± 0.2) GBq/µA. An energy of 0.84 MeV is absorbed in the CsCl layer of the target. Considering the thick target yield, a maximum activity of 320 MBq can be expected with the used beam parameters. Moreover, directly after the end of bombardment (EOB), the radionuclides chlorine-34m, cesium-131, cesium-132, barium-131m, barium-131, and barium-133m were detected. After two days of decay time for the short-lived side products, mainly chlorine-34m (approx. 5.7 GBq) and several co-produced gold isotopes in the Pt target backing, the target was worked-up to isolate the barium isotopes from the bulk cesium salt as well as the radioactive isotopes cesium-131/-132. Therefore, Sr Resin was used applying the method described in Section 3.4. A barium-131 recovery of (80 ± 8) % of the initially measured barium-131 activity (directly before the work-up) was achieved after purification. The obtained elution profile of cesium and barium, portrayed by the volume-dependent activity of barium-131 and cesium-131 is displayed in Figure 4.

Since the separation was completed, cesium was not detectable anymore in the product solution, and only barium-131 and barium-133m were found, whereby barium-133m is responsible for the formation of the isotopic barium impurity barium-133. Thereby we conclude, that our applied work-up method is applicable for removing both the bulk natural cesium and the cesium radioisotope impurities.

One of our major obstacles is the amount of barium-133 impurity, so the calculation of the barium-131/-133 ratio is necessary. Consequently, retention samples were measured three months after EOB, and the activity ratio was calculated to be approx. 7∙10^3^ to 1 for all samples. This means that about 1 kBq of barium-133 is produced for each 7 MBq of barium-131, which corresponds with the injection dose for one mouse. We assume that roughly 100 MBq of barium-131 would be needed for human imaging in the human situation, leading to a coincident injection of 15 kBq barium-133 impurity. Taking into account that more than 80% of the injected dose should have been excreted within 24 h, less than 3 kBq barium-133 remains (same range as average kalium-40-content in the human body), which is possibly important for dosimetric assessment. Barium-133 excretes via the same metabolic pathway as barium-131 due to the same physiological behavior, which reduces its amount noticeably.

### 2.3. Radiolabeling

**Figure 5:**
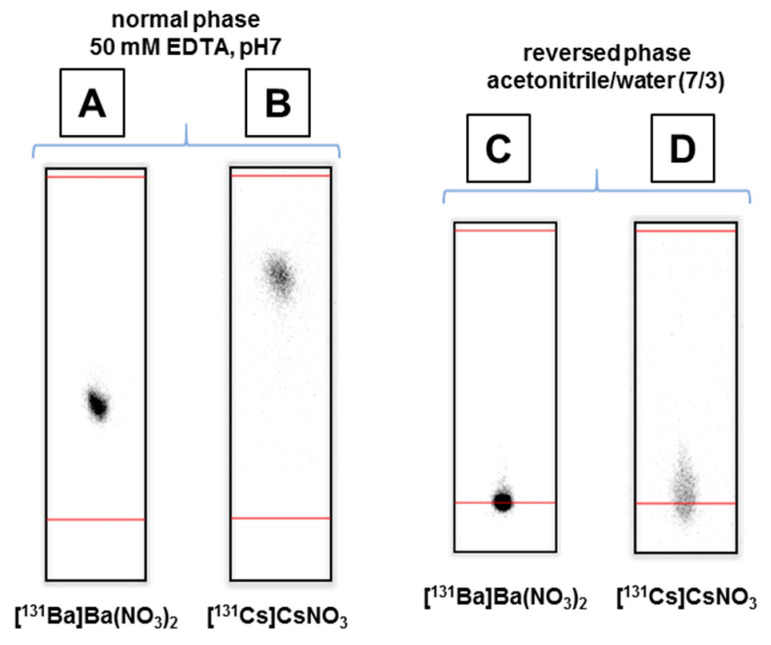
Radio-TLCs of [^131^Ba]Ba^2+^ and [^131^Cs]Cs^+^ using normal phase TLC plates (**A**,**B**) and reverse phase TLC plates (**C**,**D**).

For quality control studies, radio-TLCs were prepared with the ^131^Ba- and ^131^Cs-stock solutions to visualize the eligibility of the above-mentioned separation method in addition to the quantification via γ-spectroscopy. Therefore, two different TLC systems, reverse and normal phase (RP/NP), were chosen. The NP system is based on a silica solid phase. The free radiometal ions in the stock solutions move along with the mobile phase (50 mM EDTA) as EDTA complexes (cf. Figure 5A,B). The R*_f_* values for Ba^2+^ and Cs^+^ are determined to be 0.35 and 0.71, respectively. The pH value of the mobile phase was chosen to be 7, close to the physiological pH value, but also low enough not to form insoluble barium hydroxy species.

Additionally, a RP system was used to immobilize free ions at the baseline (cf. Figure 5C,D). Due to the high acetonitrile content of the mobile phase (70 vol%), it is optimal to separate free metal ions and the radiolabeled organic ligand, which will move along with the mobile phase.

The radiolabeling with barium-131 and cesium-131 was investigated using the complexing agent macropa, a well-known ligand from the literature. Anyhow, only a few approaches with macropa and alkaline earth metal ions have been published so far [22,23]. Showing the HPLC stability for the macropa complex is challenging (obviously depending on stability issues in highly diluted systems), which is why we have developed the above mentioned two different TLC systems, one for providing information about the complexation of free barium, the other one for showing that only one reaction took place by having only one signal at the TLC plate. The nonradioactive Ba-macropa complex was fully characterized by Wilson and co-workers [23]. Both stock solutions (^131^Ba and ^131^Cs) were used for radiolabeling, which was performed according to the conditions described in Section 3.5.

**Figure 6:**
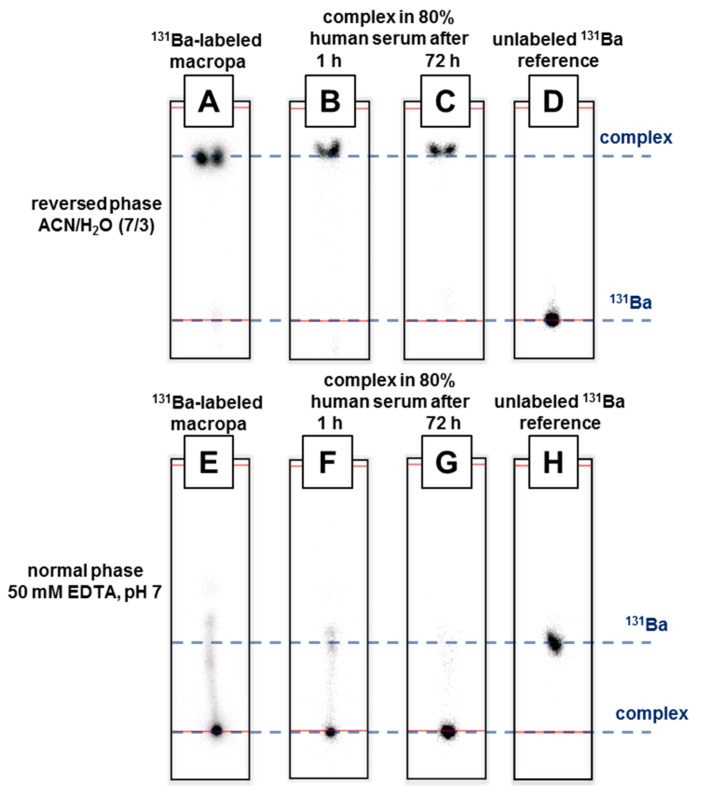
Radio-TLCs of ^131^Ba-labeled macropa (**A** and **E**) in human serum for stability testing using RP TLC (**B** and **C**) and NP TLC (**F** and **G**) and free [^131^Ba]Ba^2+^ as reference (**D** and **H**).

An efficient labeling was determined for macropa up to a concentration of 10^−4^ M. The desired ^131^Ba complex was verified by the RP and NP TLC system. Considering the high amount of the competitive EDTA chelator in the NP system and the weak acceptor and donor properties of heavy alkaline earth metals, this is a satisfying result. Due to the promising radiolabeling with macropa, the ^131^Ba-macropa complex was tested against human serum. No release of the radiometal was determined by RP TLC within three days, even at a high serum concentration of 80 vol% (cf. Figure 6A–C). Nevertheless, due to the high concentration of the competing chelator EDTA of 50 mM in the NP TLC system (cf. Figure 6E–G), a slight dissociation of the complex was observed. However, both TLC systems proved a sufficient plasma stability of the ^131^Ba-macropa complex. The labeling of macropa with cesium-131, performed under equal conditions, did not lead to any TLC-detectable complex formation.

Furthermore, adsorption studies of barium 131 onto hydroxyapatite were performed, showing a rapid and efficient binding of [^131^Ba]Ba^2+^ with >99% within 5 min, which led us to the assumption that a rapid in vivo bone uptake will occur. After seven days, this ^131^Ba-labeled hydroxyapatite sample was centrifuged and the pellet was washed twice. The released cesium-131 was detected in the washing solutions, meaning, that there is no affinity of bone-like materials against the released cesium, even if the mother radionuclide has been stably captured. In contrast, once the barium-131 was stably bound to macropa, no adsorption onto hydroxyapatite was detected. From this observation, we conclude that this could be an important finding towards use of barium-131 in a non-calcimimetic way. Using the ^131^Cs-stock solution, no interaction with hydroxyapatite was detected.

### 2.4. SPECT Imaging

**Figure 7:**
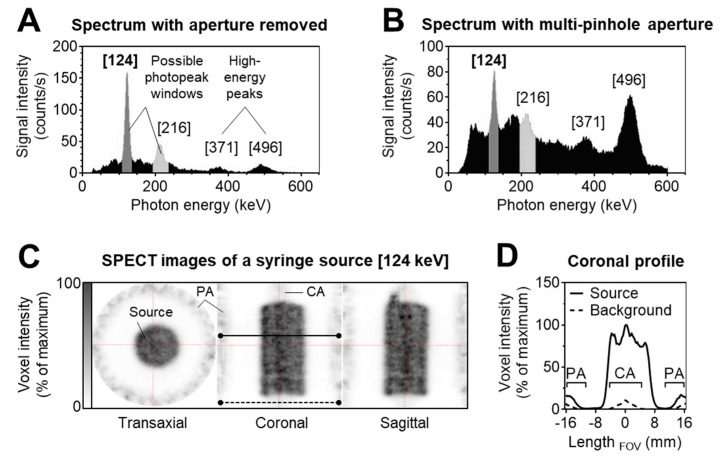
Single photon emission computed tomography (SPECT) imaging performance of barium-131. (**A**) Energy spectrum of a 0.5 MBq point source recorded without aperture showing two possible photopeaks at 124 keV and 216 keV (grey) and two high-energy peaks at 371 keV and 496 keV (black); (**B**) energy spectrum of a 3 MBq syringe source recorded with multi-pinhole aperture showing relative increase of high-energy peaks and noise (black) contaminating the desired photopeak windows (grey); (**C**) orthogonal SPECT images of a 3 MBq syringe source recorded using the 124 keV photopeak window; positions of representative axes for measurement of voxel intensity profiles; dashed line: Background zone, *n* = 6; continuous line: Source zone, *n* = 9; (**D**) voxel intensity profiles of the central of the coronal image slice shown under C; peripheral artifacts (PA) occurring in the periphery of the field of view (FOV); central artifacts (CA) occurring along the central anterior-posterior axis of the FOV.

To select suitable photon energy windows for SPECT imaging of barium-131, an energy spectrum was recorded without multi-pinhole collimation, showing two suitable photopeaks at 124 keV and 216 keV, as well as two prominent high-energy peaks at 371 keV and 496 keV (Figure 7A). However, installing the aperture with the multi-pinhole collimators, providing special information, decreased the relative heights of the suggested photopeaks compared to the high-energy peaks, and added considerable noise to the suggested photopeak windows (Figure 7B). Most likely, these effects occurred since the attenuation of the high-energy photons in the collimator material is much less compared to the lower-energy photons within the photopeak windows.

Such effects have already been reported for iodine-123, a SPECT radionuclide that, besides the primary photopeak at 159 keV (83%), also emits high-energy photons, most abundantly at 529 keV (1.4%). Many of these high-energy photons are assumed to pass through the crystals of the SPECT camera and undergo Compton scattering from the components adjacent to the crystals (e.g., photo-multiplier tubes and associated electronics). These inelastically scattered photons are then detected by re-entering the crystal with a lower energy contaminating the projection data recorded within the photopeak windows [29].

For barium-131, the percentage of high-energy photons, in particular at 371 keV (14%) and 496 keV (48%), and their contribution to image blurring and quantification errors is much higher compared to iodine-123. Due to the high intensity of noise interfering in particular with the 216 keV photopeak window, investigations on SPECT imaging of barium-131 were performed, recording only the 124 keV photopeak window.

SPECT imaging of a cylindrical syringe source filled with an aqueous solution of [^131^Ba]Ba(NO_3_)_2_ showed that signals at the position of the source were surrounded by noise-derived artifacts accumulating in the periphery of the field of view (FOV) (peripheral artifacts) as well as along the central anterior–posterior axis of the FOV (central artifacts) (Figure 7C). Such artifacts appear because the physical model used in the Tera-Tomo 3D reconstruction algorithm is inconsistent with the contaminated barium-131 projection data. Since there is less measured information content in the periphery of the FOV, it is more affected by this inconsistency, hence, stronger artefacts appear. Quantitative analyses showed that 52% of total signal intensity was located at the position of the source, whereas the remaining 48% were located within the ring of peripheral artifacts. Furthermore, voxel intensity profiles, measured across the central coronal slice image, showed inhomogeneity across the center of the source with variation up to 25%, due to overlap with the central artifacts (Figure 7D). Moreover, the correlation analysis showed a significant positive relationship between voxel intensities of the source and the voxel intensities of peripheral artifacts (*r*_p_ = 0.79, *p* < 0.001). These results demonstrate that SPECT imaging enables visualizing barium-131, with considerable limitations due to artifacts, deriving from high-energy photon emission. Use of high-energy collimators may allow for overcoming these limitations in future investigations. Until then, quantitative investigations on barium-131 in vivo rely on alternative techniques, such as activity measurement in tissue samples.

**Figure 8:**
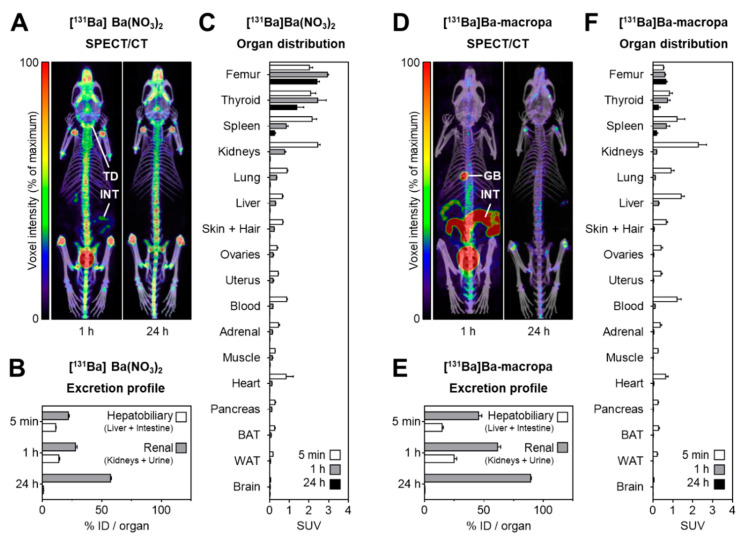
Distribution of [^131^Ba]Ba(NO_3_)_2_ and [^131^Ba]Ba-macropa in mice. Small animal SPECT images were recorded using the 124 keV photopeak window, post-processed for removal of peripheral artifacts, fused with computed tomography (CT) images, and displayed as maximum intensity projections; scaling of voxel intensities was matched with 1-h image; (**A**) SPECT/CT fusion images of [^131^Ba]Ba(NO_3_)_2_ in a mouse 1 h and 24 h after injection; (**B**,**C**) excretion profile and organ distribution of [^131^Ba]Ba(NO_3_)_2_ in mice 5 min, 1 h, and 24 h after injection (*n* = 4); (**D**) SPECT/CT fusion images of [^131^Ba]Ba-macropa in a mouse 1 h and 24 h after injection; (**E**,**F**) excretion profile and organ distribution of [^131^Ba]Ba-macropa in mice; 5 min, 1 h, and 24 h after injection (*n* = 4); (BAT) brown adipose tissue; (GB) gall bladder *; (ID) initial dose; (INT) intestine; (TD) thyroid/parathyroid *; (WAT) white adipose tissue (* activity in these organs was not measured separately).

Despite the limitations regarding the SPECT imaging performance of barium-131, small animal SPECT/CT (computed tomography) imaging followed by removal of peripheral artifacts during post-processing provided sufficient image quality for visualizing the distribution of [^131^Ba]Ba(NO_3_)_2_ and ^131^Ba labeled macropa in mice (Figure 8A,D). After intravenous injection of [^131^Ba]Ba(NO_3_)_2_ in mice, small animal SPECT/CT imaging showed an accumulation of barium-131 in the entire skeleton that established within 1 h after injection, and was still present after 24 h (Figure 8A). Barium-131 showed the highest activity concentration in particular in bone regions with high metabolic activity such as epiphyses, vertebral bodies, and the upper jaw. Activity measurement in tissue samples confirmed the distribution pattern of [^131^Ba]Ba(NO_3_)_2_ observed in SPECT images showing that barium-131 was excreted predominantly via the renal pathway (57% ID/organ within 24 h) and, to a lesser extent, also via the hepatobiliary pathway (14% ID/organ within 1 h) (Figure 8B).

Organ distribution showed a rapid blood clearance and confirmed a rapid bone uptake of [^131^Ba]Ba(NO_3_)_2_ as occurred in the femur within 5 min (SUV = 2.02), increased until 1 h (SUV = 2.94), and decreased again until 24 h (SUV = 2.41) (Figure 8C). Activity levels of [^131^Ba]Ba(NO_3_)_2_ accumulating in bones of mice are in accordance with previous reports [2]. Similar to barium-131, bone accumulation in mice has also been reported for radium-223 after injection of [^223^Ra]RaCl_2_, with highest activity concentrations observed within the inorganic bone matrix of the epiphyses [30]. For subsequent removal of barium-131 from the skeleton, several mechanisms have been discussed previously, such as physiological bone resorption, as well as successive replacement of barium by calcium followed by diffusion of the released barium ion through the calcified matrix, with some prospect of re-entry into blood vessels [31,32].

A rapid uptake of barium-131 is also observed in the thyroid within 5 min (SUV = 2.08), increased until 1 h (SUV = 2.44), and decreased again until 24 h (SUV = 1.38). This uptake possibly occurs through the sodium iodine symporter (NIS) system. Similar to [^131^Ba]Ba(NO_3_)_2_, thyroid accumulation has been reported for radium-226 in humans [33]. Otherwise, barium-131 may also have accumulated in parathyroid, a pair of small but important endocrine glands on both sides of the thyroid that synthesize parathyroid hormone regulating mainly phosphoric calcium metabolism. Parathyroid glands have been reported to accumulate isotopes of cesium, strontium, and iodine [34]. However, the small organ size in mice does not allow for measuring the activity in tissue samples of the parathyroid separately.

After intravenous injection of ^131^Ba-labeled macropa in mice, small animal SPECT/CT imaging showed rapid passage of the radiolabeled complex through gallbladder and intestine within 1 h after injection, followed by only some accumulation in the skeleton and bigger joints, detectable after 24 h (Figure 8D). Activity measurement in tissue samples confirmed the distribution pattern observed in SPECT images showing that, compared to [^131^Ba]Ba(NO_3_)_2_, significantly (*p* < 0.001) higher amounts of ^131^Ba-labeled macropa were excreted via the renal pathway (89% ID/organ within 24 h) and also via the hepatobiliary pathway (25% ID/organ within 1 h) (Figure 8E).

Organ distribution showed rapid clearance of ^131^Ba-labeled macropa from blood and significantly (*p* < 0.001) lower accumulation in bones compared to [^131^Ba]Ba(NO_3_)_2_, as occurred in the femur within 5 min (SUV = 0.53) and slightly increased within 24 h (SUV = 0.65) (Figure 8F). Furthermore, injection of ^131^Ba-labeled macropa resulted in significantly (*p* < 0.001) lower accumulation of barium-131 in the thyroid or parathyroid compared to [^131^Ba]Ba(NO_3_)_2_. These results demonstrate a striking difference of biodistribution behavior between ^131^Ba-labeled macropa and free [^131^Ba]Ba^2+^. The radiolabeled complex accumulates barely in bones and the thyroid/parathyroid. Especially the femoral uptake with 2.19%IA/g after 24 h for [^133^Ba]Ba-macropa is comparable with [^223^Ra]Ra-macropa (1.6%IA/g after 24 h), shown previously [24]. These findings lead to the conclusion that barium-131 is a perfect radionuclide for a matched pair with radium-223/-224.

## 3. Materials and Methods

### 3.1. Target Preparation

**Figure 9:**
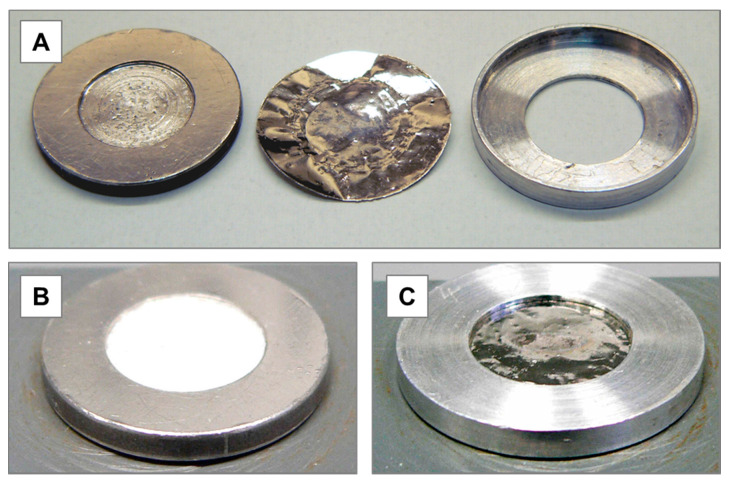
(**A**) Pt-target disk, Pt-foil, and Al-holder for the irradiation; (**B**) open ^nat^CsCl target after pressing and before closing with Pt foil; (**C**) complete target after 4 h of irradiation.

A platinum disk (2 mm thickness, 22 mm diameter) was used as target backing, offering a 0.2 × 12 mm centered circular deepening (Figure 9A). 80 mg of anhydrous cesium chloride were pressed into this cavity (Figure 9B) using a manual hydraulic press (laboratory press PE-MAN) with a compressive load of 60–80 kN. The target was covered with a platinum foil (10 µm thickness, 20 mm diameter) and locked by an aluminum holder. Due to the hygroscopic properties of cesium chloride, the target was prepared no later than 24 h before irradiation. To avoid high gamma doses from several co-produced short-lived radionuclides, the irradiated target (Figure 9C) was stored for 48 h after EOB. The target material was then dissolved in 0.5 mL of HNO_3_ (3 M) and worked up as described below.

### 3.2. Irradiation Conditions

The irradiation of the targets was performed using the TR-FLEX (ACSI) cyclotron at HZDR. The prepared targets were inserted into the 90° solid target holder. The proton beam was extracted with an energy of 30 MeV and a current of 30 µA. A 0.7 mm thick energy degrader system, made of aluminum, was installed in front of the 90° target holder so that the proton energy on the target was reduced to 27.5 MeV. The proton beam was less focused on the target disk to ensure a homogeneous irradiation of the whole target surface and to prevent spots with high beam power. Nearly half of the extracted ion current hit the circular collimator and the 4-sector-aperture in front of the 90° solid target holder. The targets were irradiated with ion currents of (15 ± 1) µA for 4 h to achieve an accumulated charge of (60 ± 4) µAh.

### 3.3. Determination and Quantification of Radionuclides

Radioactivity measurements were performed with an energy and efficiency calibrated high-purity germanium detector (HPGe, CryoPulse5, Canberra, Australia) for analytic quantification. Experimental samples (200 µL) were counted for 3 min using a dynamic energy window of 1–1750 keV. The recorded data were analyzed using the Genie2000 software (V.3.4.1).

### 3.4. Barium-131 and Cesium-131 Separation

**Figure 10:**
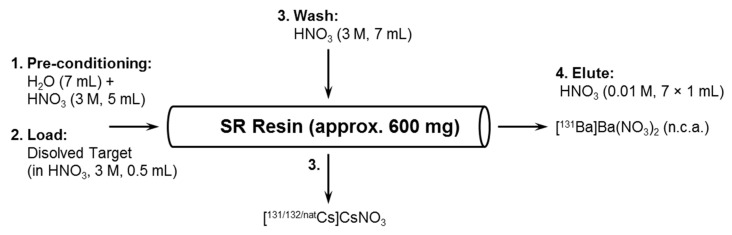
Method scheme for the separation of barium-131 from cesium impurities.

SR Resin (TRISKEM, Bruz, France, 600 mg) was used as solid phase, packed in a 6 mm diameter reservoir, pre-conditioned with deionized H_2_O (7 mL) and HNO_3_ (3 M, 5 mL). After a period of 48 h, the target was dissolved in HNO_3_ (0.5 mL; 3 M). The obtained clear solution was passed through this cation exchange resin. After loading the dissolved target, the matrix was rinsed with HNO_3_ (3 M, 7 mL k′ = 15 for Ba^2+^, k′ = 0.8 for Cs^+^) to elute the cesium content. Subsequently, diluted HNO_3_ (0.01 M, 7 × 1 mL; k′ < 1 for Ba^2+^) was used to elute [^131^Ba]Ba(NO_3_)_2_ (cf. Figure 10). The maximum of activity was obtained in volume fraction at milliliters 4 and 5 (cf. Figure 4). These two milliliters were combined and used as stock solution without further purification.

**Figure 11:**
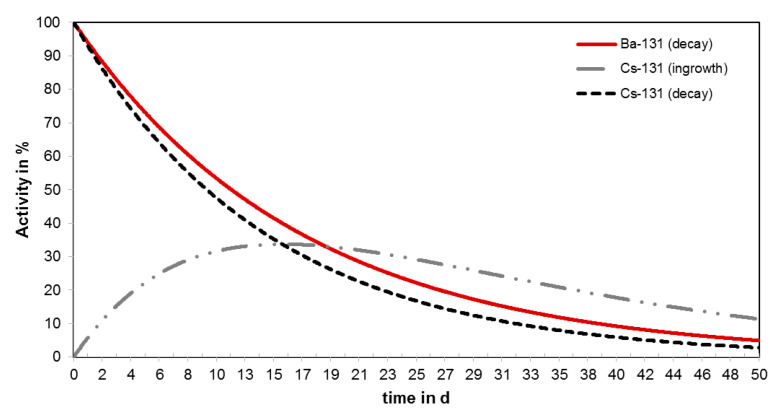
Calculated decay scheme of barium-131 and cesium-131.

After a period of 10 d, a sufficient amount of the daughter nuclide cesium-131 was obtained from the [^131^Ba]Ba(NO_3_)_2_-stock solution (cf. Figure 11). The separation of cesium-131 was performed under the above-mentioned conditions (cf. Figure 10). The purified ^131^Cs-stock solution was obtained combining the first 5 milliliters of the 3rd step (HNO_3_, 3 M). The mother nuclide barium-131 can be recovered by step 4 (HNO_3_, 0.01 M).

### 3.5. Radiolabeling and Reaction Control

Radiolabeling was performed using the chelator macropa, which was synthesized in a two-step procedure according to the literature [35]. All chemicals were purchased from commercial sources and used without further purification. Stock solutions of macropa were prepared in advance (10^−2^ M, 10^−3^ M, 10^−4^ M). 10 µL of any chelator stock solution were added to 85 µL of 0.2 M ammonium acetate buffer (pH 6). 100 kBq (V = 5 µL) of [^131^Ba]Ba(NO_3_)_2_ in 0.01 M HNO_3_ were finally added to the buffered ligand solution and the samples were mixed for one hour at ambient temperature in a thermomixer. Reaction control was performed with an aliquot of 2 µL by thin layer chromatography (radio-TLC), using two different systems. For the normal phase (NP), TLC plates (Silica Gel 60, F_254_, Merck, (Darmstadt, Germany) cut to 10 × 2 cm) and 0.05 M EDTA (in water, pH 7, adjusted with NaOH) was used as the mobile phase. For the reversed phase (RP), Alugram (Nano SIL CN/UV254, Machery-Nagel, (Düren, Germany) cut to 8 × 2 cm) TLC plates and a mixture of acetonitrile and water 70/30 *v*/*v* was used as the mobile phase. The radio-TLCs were scanned using an instant phosphor imager (Amersham Typhoon scanner, GE Healthcare Bio-Sciences Corp., Marlborough, MA, USA). The data was analyzed using the AIDA software (V. 5.1 SP4).

The serum stability tests of the ^131^Ba-macropa complex were performed as follows. The ^131^Ba-labeled macropa solution (100 µL, final macropa conc. 10^−3^ M) was added to human serum (400 µL, Biochrom, Cambridge, UK). The sample was mixed in a thermomixer at 37 °C for 3 d. Aliquots of 2 µL were taken after 10 min, 1 h, 6 h, 24 h, and 72 h, and were directly spotted on TLC plates (NP and RP) and measured as described above. The adsorption studies were accomplished by rapidly mixing 5 MBq of [^131^Ba]Ba(NO_3_)_2_, [^131^Cs]CsNO_3_, or 1 MBq of ^131^Ba-macropa complex and 20 mg of hydroxyapatite in 1.2 mL of water. The samples were incubated in an overhead shaker at ambient temperature overnight. Aliquots were taken after centrifuging the samples, and washing the pellet with water twice. Activity analyses regarding the pellet/supernatant-ratio were carried out using a HPGe detector.

### 3.6. Animal Experiments

Animal experiments were performed at HZDR according to the guidelines of German Regulations for Animal Welfare and have been approved by the local Animal Ethics Committee for Animal Experiments (Landesdirektion, Dresden, Germany). Investigations were performed using female athymic nude mice (Rj:NMRI-Foxn1nu/nu, Janvier Labs, Le Genest-Saint-Isle, France), between 12 and 14 weeks old, housed in a pathogen-free facility. Animals received one intravenous injection (0.1–0.2 mL; tail vein) of [^131^Ba]Ba(NO_3_)_2_ diluted in 0.01 M HNO_3_, pH 6, or ^131^Ba-macropa (0.1–0.2 mL in 0.2 M ammonium acetate; pH 6), respectively. For in vivo imaging, anesthesia was induced and maintained with inhalation of 10 vol% desflurane (Baxter, Deerfield, IL, USA) in 30 vol% oxygen air, and body temperature of the animals was maintained at 37 °C. After the last imaging examination and during biodistribution studies, animals were sacrificed using CO_2_ inhalation and cervical dislocation, and organs were excised for further analyses.

### 3.7. SPECT Imaging

SPECT and X-ray computed tomography (CT) were performed using the dedicated small animal nanoScan SPECT/CT scanner (Mediso, Budapest, Hungary). Uniformity and energy calibration were performed without collimators recording the photon emission of a [^131^Ba]Ba(NO_3_)_2_ point source (0.5 MBq in 20 µL of 0.01 M HNO_3_) within the 20% energy windows of the 124 keV and 216 keV photopeaks, respectively. SPECT imaging was performed using a standard aperture for mouse imaging (APT62) consisting of four M3 multi-pinhole collimators providing a 30 × 30 mm transaxial field of view (FOV). Photon emission was recorded within the 20% energy window of the 124 keV photopeak. CT images were captured at a peak kilovoltage of 35 kVp and were used for attenuation correction and for anatomical referencing. Imaging performance of barium-131 was tested recording a cylindrical syringe source (3 MBq of [^131^Ba]Ba(NO_3_)_2_ in 3 mL of 0.01 M HNO_3_) with a frame time of 400 s (total scan time: 4.5 h). Small animal SPECT/CT imaging in mice was performed at 1 h and 24 h after i.v. injection of [^131^Ba]Ba(NO_3_)_2_ (6.2 MBq in 0.2 mL of 0.01 M HNO_3_, pH 6, A_m_ = 420 GBq/µmol, n.c.a.), or ^131^Ba-labeled macropa (6.7 MBq in 0.2 mL of 0.1 M ammonium acetate, pH 6, A_m_ = 83 MBq/µmol) with a frame time of 60 s (total scan time: 1.5 h), respectively. Projection data were reconstructed using the Tera-Tomo™ 3D high dynamic range algorithm (resolution: 128; iterations: 48; subset size: 4), applying corrections for decay, scatter, and attenuation.

Images were post-processed and analyzed using ROVER software (ABX GmbH, Radeberg, Germany). In SPECT images of the syringe source, total voxel intensities were compared within volumes of interest (VOIs) including the entire FOV or the position of the source at a threshold of 0%, respectively. Profiles of voxel intensities were measured across the central coronal slice image of the syringe source comparing background zones (*n* = 6) and source zones (*n* = 9). In SPECT images of mice, peripheral artifacts were removed during post-processing. Images were fused with CT images and displayed as maximum intensity projections (MIPs) at equal scaling of voxel intensities.

### 3.8. Biodistribution

Biodistribution of [^131^Ba]Ba(NO_3_)_2_ (400 kBq in 0.1 mL of 0.01 M HNO_3_, pH 6, A_m_ = 420 GBq/µmol, n.c.a.) and ^131^Ba-labeled macropa (400 kBq in 0.1 mL of 0.1 M ammonium acetate, pH 6, A_m_ = 83 MBq/µmol) in tissue samples of mice was measured at 5 min, 1 h, and 24 h after injection. At each time point, animals were sacrificed (*n* = 4), and organs and tissues were excised and weighted. Activity was determined using the gamma counter Wizard (PerkinElmer, Waltham, MA, USA) and corrected for decay. Excretion via hepatobiliary (liver + intestine) and renal (kidneys + urine) pathways were reported as % initial dose (ID)/g organ. Organ distribution was reported as standardized uptake value (SUV = [MBq accumulated/g tissue]/[MBq injected/g body weight]).

### 3.9. Statistical Analyses

Statistical analyses were performed using Prism 8.0 (GraphPad Software, San Diego, CA, USA). Significance of differences was tested using analysis of variation (ANOVA) applying Sidak’s multiple comparison post-hoc test. Differences were considered significant at *p* values < 0.05. Statistical relationships were analyzed using Pearson’s correlation test and reported as correlation coefficient, *r*_p_.

## 4. Conclusions

We here show for the first time the in vivo biodistribution behavior of ^131^Ba-labeled macropa in comparison with free [^131^Ba]Ba^2+^ by means of small animal SPECT/CT. For that purpose, within this work, we established a straight forward cyclotron production of 190 ± 26 MBq non-carrier added barium-131 via the ^133^Cs(*p*,*3n*)^131^Ba nuclear reaction using 27.5 MeV proton beams. A rather low isotopic barium-133 impurity of 0.01% regarding the activity of barium-131 was determined. The simple, but very efficient, purification of the irradiated CsCl target was performed using the SR Resin, providing the [^131^Ba]Ba^2+^ as diluted barium nitrate, perfectly suitable for labeling experiments and in vivo applications. Besides barium-133, no other radionuclide impurities were detected. Production and purification processes are potentially feasible in accordance to GMP guidelines. The activity amount received via one irradiation already allows whole body imaging of two humans.

However, at this stage, the produced barium-131 is more suitable for preclinical studies than for common application. In perspective, a further scale-up and optimization of the irradiation procedure, especially the usage of a 30° target holder, higher beam current, and longer irradiation times will lead to substantially higher production yields combined with a decrease of barium-133 at the same time.

A convenient kit-like labeling of barium-131 with the chelator macropa was performed at room temperature. Small animal SPECT/CT imaging and biodistribution studies were performed using [^131^Ba]Ba(NO_3_)_2_ and ^131^Ba-labeled macropa in healthy mice. The results demonstrate that barium-131 in its ionic form is applicable as bone targeting radiotracer. In general, reliable quantitative image analysis will require further detector and collimator optimization to prevent image artifacts and other side effects caused by high-energy photons. The in vivo behavior of ^131^Ba-labeled macropa was examined and showed a rapid blood clearance and significantly lower bone uptake. However, a not negligible bone uptake was observed. For this purpose, the development of improved chelators is indispensable in the next steps. Once a more stable complexing agent for barium-131 is found, active targeting via biomolecules will enable long-term imaging of different tumor entities without unintended bone enrichment. We conclude that, especially due to the matching properties of barium and radium, barium-131 will unroll its importance as the imaging counterpart to radium-223, a radionuclide of great interest for future applications in targeted alpha therapy.
